# Isolated Positive Treponemal Test in Pregnancy and Placental Abnormalities Without Confirmed Syphilis Infection: A Case Report

**DOI:** 10.1155/crdi/7069854

**Published:** 2025-10-13

**Authors:** Elizabeth Stiles, Margaret L. Aldrich, Margret S. Magid, Caitlin Otto, Andrew Rubenstein

**Affiliations:** ^1^Department of Obstetrics and Gynecology, NYU Langone Health, New York, New York, USA; ^2^Department of Pediatrics, NYU Langone Health, New York, New York, USA; ^3^Department of Pathology, NYU Langone Health, New York, New York, USA; ^4^Microbiology Laboratory, NYU Langone Health, New York, New York, USA

**Keywords:** congenital syphilis, false-positive treponemal test, placental pathology, pregnancy, syphilis screening

## Abstract

Syphilis screening during pregnancy is essential to prevent congenital syphilis, yet diagnostic challenges arise when clinical presentation, serologic results, and pathologic examination are discordant. We report the case of a 39-year-old pregnant patient with a reactive enzyme immunoassay (EIA) at delivery, despite prior nonreactive syphilis serologies and a negative confirmatory test postpartum. Placental examination revealed multiple intervillous abscesses and chronic villitis, raising concern for congenital syphilis. However, immunohistochemistry (IHC) for *Treponema pallidum* yielded conflicting results across laboratories. Despite the lack of confirmed maternal infection, the neonate underwent a full congenital syphilis evaluation and received penicillin treatment. This case highlights the complexities of interpreting isolated positive treponemal tests, the limitations of placental pathology in syphilis diagnosis, and the need for standardized management algorithms to prevent misclassification, overtreatment, and undue emotional and healthcare burden. Interdisciplinary communication and rapid confirmatory testing are critical to optimizing maternal and neonatal outcomes.

## 1. Introduction

Syphilis screening in pregnancy is critical for the prevention of adverse maternal and fetal outcomes, including congenital syphilis. Given the rising rates of congenital syphilis, the American College of Obstetricians and Gynecologists (ACOG) recommends universal serologic screening for syphilis at the first prenatal visit, in the third trimester, and at delivery regardless of risk [1, 2]. The diagnostic algorithm relies on treponemal (e.g., EIA, *T*. *pallidum* particle agglutination [TPPA], chemiluminescent immunoassay [CLIA]) and nontreponemal (e.g., Rapid Plasma Reagin [RPR], Venereal Disease Research Laboratory [VDRL]) tests. As treponemal test automation improves, the reverse screening algorithm (treponemal test first, then confirmation with a nontreponemal test and, when necessary, confirmation with another treponemal test) is becoming more widely adopted [3, 4].

An isolated positive syphilis screening test at delivery triggers an expedited workup, including repeat serologic testing, clinical assessment of both birthing parent and newborn and placental histopathology to determine the risk of congenital infection [5]. False positive treponemal and nontreponemal tests can arise from various conditions, including autoimmune disorders, acute viral infections, recent vaccinations, injection drug use, and pregnancy [6–9]. The prozone effect during peak viremia can lead to false-negative nontreponemal tests in high-titer infections due to antibody excess interfering with agglutination. In such cases, serial dilution of the specimen may be required to unmask true reactivity [10, 11]. Placental examination may inform diagnosis of in utero syphilis infections. However, the pathological findings are not specific to syphilis and frequently require further specialized studies and clinical correlation [12].

This case report highlights the diagnostic challenges of syphilis in pregnancy, including interpretation of an isolated reactive EIA at delivery and abnormal placental findings in the absence of confirmed infection. A systematic approach to diagnostic uncertainty is essential to avoid delay in diagnosis, overtreatment, unnecessary neonatal evaluation, and misclassification of congenital syphilis cases.

## 2. Case Report

A 39-year-old G4P1021 Hispanic female with a history of asthma, seasonal allergies, obesity, and marginal cord insertion presented to a large urban academic hospital for scheduled induction of labor at 39 weeks' gestation (indicated for gestational diabetes mellitus type A2). She had received regular prenatal care at an affiliate practice starting in the first trimester. During pregnancy, her medications included metformin (500 mg twice daily), aspirin (81 mg daily for preeclampsia prophylaxis), prenatal vitamin, cholecalciferol, ferrous sulfate, fexofenadine, and docusate sodium. The patient denied any significant gynecologic history including sexually transmitted infections (STIs). Routine STI screenings were negative, including nonreactive RPR in the first (collected at 7 weeks and 4 days' gestation) and early third (collected at 27 weeks and 5 days' gestation) trimesters. CLIA conducted later in the third trimester (at 35 weeks and 5 days' gestation) was also nonreactive.

Upon presentation for induction at 39 weeks' gestation, the patient's syphilis screening tests were repeated. The patient had an uncomplicated induction of labor with misoprostol, oxytocin, artificial rupture of membranes, and amnioinfusion (in the setting of persistent category II fetal heart rate tracings). She underwent a normal vaginal delivery at 39 weeks and 1 days' gestation with epidural of a healthy appearing (APGARS 9 at 1 and 5 mins) male weighing 3204 g (7 pounds, 1 ounce). The delivery was complicated by periurethral and first-degree lacerations.

**Figure 1:**
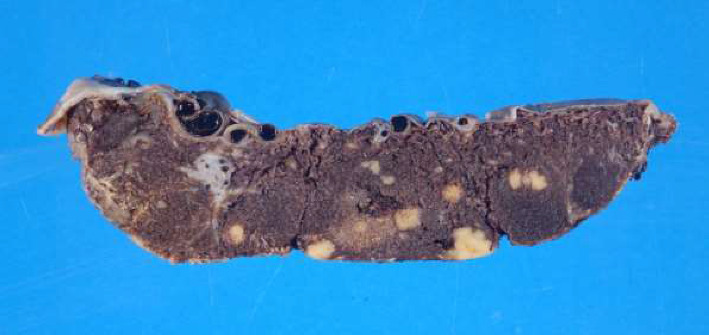
A cut section through the placenta with the fetal surface at the top and the maternal surface at the bottom of the image. Multiple yellow-white nodules are scattered throughout the dark red villous parenchyma.

**Figure 2:**
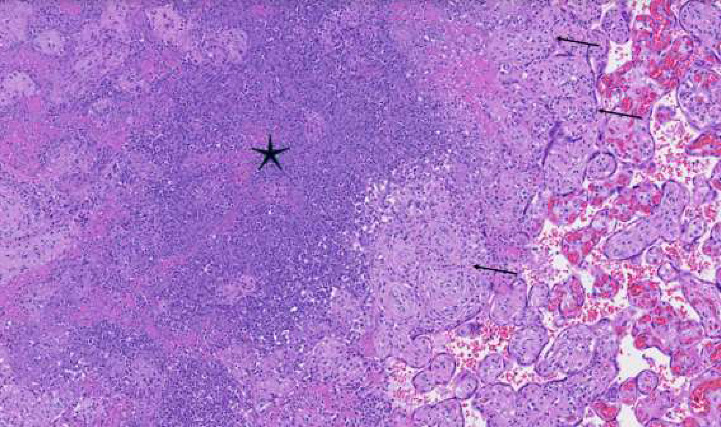
A microscopic section of one of the placental lesions shows central abscess formation with neutrophilic debris and necrotic villi (star), rimmed by villi showing chronic inflammation (chronic villitis) (arrows). Hematoxylin and eosin stain, 10x.

Syphilis screening results revealed reactive EIA. Follow-up RPR and TPPA were nonreactive. The placenta underwent pathologic review in the setting of GDMA2. The placental weight was 387 g (10th percentile). Numerous soft yellow-white nodular lesions were evident on cut section, ranging in diameter from 0.2 to 2.1 cm (Figure 1). Histological review revealed that the lesions were multiple intervillous abscesses, focally rimed by small clusters of chronic villitis and avascular fibrotic villi, the latter of which likely resulted from inflammatory obstruction to proximal fetal blood flow (Figure 2). Viral inclusions were not identified. Gram stain revealed extensive karyorrhectic debris but no definitive bacterial organisms. Evaluation for herpes simplex virus by immunohistochemistry was negative. Immunostain performed at the hospital on formalin-fixed paraffin-embedded tissue was interpreted as positive, although abundant artifactual stain precipitate was noted. A repeat immunostain performed at a reference laboratory was negative. Acute chorioamnionitis was not identified, and the umbilical cord was unremarkable.

The patient was discharged on postpartum day 1 with prophylactic Lovenox (40 mg daily for 14 days; RCOG 2). Given the results of placental pathology, the infant was admitted on day of life 25 to the children's hospital for congenital syphilis treatment with penicillin after a complete evaluation, inclusive of a lumbar puncture. The infant was ultimately lost to follow up. The patient was counseled on outpatient repeat testing and referred to infectious disease specialists. Approximately 5 weeks after delivery, the patient's confirmatory CLIA was negative.

## 3. Discussion

This patient's inconsistent clinical presentation, serologies, and placental pathology highlight the diagnostic uncertainties faced by obstetricians, pathologists, and pediatric infectious disease specialists treating potential syphilis in pregnancy. A systematic approach is crucial when faced with discordant syphilis serologies at delivery, particularly given real-time constraints on decision-making. In this case, the patient was asymptomatic, had multiple prior nonreactive syphilis tests throughout pregnancy, and repeat testing was negative. Given no documented prior syphilis infection or treatment and negative CLIA approximately 5 weeks postpartum, the reactive EIA was most likely a false-positive rather than an active or untreated infection. Possible causes of a false-positive treponemal test in this specific case include pregnancy-related immunologic changes or an underlying nonsyphilitic condition affecting serologic reactivity [6–9]. While the prozone effect can result in false negative nontreponemal tests, the patient's nonreactive confirmatory CLIA makes this unlikely [10, 11].

Particularly in cases with discordant serologies, placental examination may aid in diagnosis. Examination of the placenta in cases of congenital syphilis often features placentomegaly (although not observed in this patient), accompanied by a histological triad of enlarged hypercellular and frequently immature villi, proliferative vascular changes of fetal blood vessels, and chronic (with or without acute) villitis [13]. Frank villous abscess formation has been described in ∼10% of cases of congenital syphilis [14, 15]. However, histology is neither specific nor highly sensitive for congenital syphilis [12, 16].

Chronic villitis is typically seen with hematogenous TORCH-type infections. In this patient, the pattern of chronic active villitis favored an infectious etiology following hematogenous spread. However, the unusual combination of acute and chronic inflammation in the villi raised consideration of less common hematogenously-disseminated pathogens, such as syphilis and herpes viruses [15]. Given rising rates of congenital syphilis, the pathological appearance of this placenta warranted ruling out this infection. Since histopathology is not pathognomonic for congenital syphilis, demonstration of the organism in tissues is required for definitive pathological diagnosis. However, the spirochetes are typically rare. Warthin-Starry silver stain is insensitive and challenging to interpret.

However, IHC for *T. pallidum* is highly specific, but sensitivity has been reported to range between 38% and 74% [14, 17]. In general, the IHC procedure is subject to many technical pitfalls that can affect interpretation and lead to conflicting results in different laboratories, as observed in this case. IHC is performed on slides prepared from formalin-fixed, paraffin-embedded blocks of tissue. Variables in tissue fixation, preservation, and embedding of tissue will affect antigen expression before the slides are cut for IHC. The technical procedure for IHC involves many steps that may vary from one laboratory to another, including the specific antibody selected (with differing sensitivity, specificity and dilution), antigen retrieval methods, and chromogen stains for visual recognition [18, 19]. False positive interpretations may occur when aberrant deposits of formalin or immunostain precipitate on the slide. In fact, previous reviews of placental IHC findings in congenital syphilis have described “dirty” backgrounds in multiple laboratories that complicated interpretation [15, 17]. Polymerase chain reaction (PCR) has also been reported as a diagnostic tool of high specificity but variable sensitivity (26%–75%) [17]. Definitive pathological diagnosis in this patient thus remained elusive.

This case also presents a conundrum for the pediatrician and/or pediatric infectious disease provider. A negative nontreponemal test result for the birthing parent at delivery does not rule out the possibility of the infant having congenital syphilis, although such a situation is rare [20]. Given the placental evaluation was ambiguous, pediatric infectious disease specialists recommended the neonate undergo a full congenital syphilis evaluation, including lumbar puncture, as well as penicillin treatment [20]. Effective communication between obstetricians, pediatricians, and pathologists is critical to ensuring appropriate neonatal management. Furthermore, rapid turnaround times (24–48 h) for newborn RPR testing, as mandated by New York State in May 2024, can help streamline clinical decision-making [21]. While erring on the side of treatment is often prudent in potential congenital syphilis cases, overtreatment poses risks. Unnecessary neonatal evaluation and antibiotic administration may contribute to antimicrobial resistance, healthcare costs, and parental anxiety from prolonged hospitalization and invasive testing. This case underscores the need for appropriate identification and management both prenatally and at delivery, given that the infant was subsequently lost to follow-up.

Continued universal syphilis screening in pregnancy is critical given the rising incidence of congenital syphilis. Teaching obstetric teams how to navigate discordant test results in pregnancy at various diagnostic algorithm branchpoints in real-time—including when to escalate care, perform serial dilutions, and involve pathologists and pediatric infectious disease specialists—may improve patient outcomes. A systematic, interdisciplinary approach is essential to minimize misclassification, prevent overtreatment, and optimize maternal and neonatal health.
